# A Pilot Study of Five Types of Maximum Hand Strength among Manufacturing Industry Workers in Taiwan

**DOI:** 10.3390/ijerph16234742

**Published:** 2019-11-27

**Authors:** Victor Ei-Wen Lo, Yi-Chen Chiu, Hsin-Hung Tu, Chien-Wei Liu, Chi-Yuang Yu

**Affiliations:** 1 Department of Occupational Safety and Health, China Medical University, Taichung City 40402, Taiwan; u101014303@cmu.edu.tw; 2 Department of Industrial Engineering and Management, Hsiuping University of Science and Technology, Taichung City 41280, Taiwan; josephdu@hust.edu.tw; 3 Department of Industrial Engineering and Engineering Management, National Tsing Hua University, Hsinchu City 30013, Taiwan; elvisliu07@gmail.com (C.-W.L.); cyyu@ie.nthu.edu.tw (C.-Y.Y.)

**Keywords:** hand strength, grip, lateral pinch, palmar pinch, thumb press, ball of thumb

## Abstract

*Background:* The purpose of this study is to collect five types of maximum hand strength among workers in the manufacturing industry in Taiwan. *Methods:* This study is a cross sectional study with a stratified and convenient sample of workers on the production line in manufacturing industries in Central Taiwan. In total, we recruited 198 healthy subjects to participate in this study. Five types of hand strength were measured in both hands three times with 3 min rests between trials. *Results:* The strength of females for these five types of hand exertions were 52.0% to 67.6% of the strength of males (*p* < 0.001). For both genders, there was a main effect for the types of hand strength for the right hand (*p* < 0.001) and the left hand (*p* < 0.001). In general, the hand strength in U.S. and EU countries was 1.2 to 1.7 times greater than the strength among the three types of hand exertions in this study. *Conclusion:* These results can be used to evaluate the musculoskeletal burdens on the upper extremities in the manufacturing industry and could also be used for tool and job design and job modifications.

## 1. Introduction

Work-related upper limb musculoskeletal disorders (WRULDs) are a significant problem in many countries and also result in critical costs, especially in manufacturing industries. WRULDs include injuries on the tendons, tendon sheath/synovium, paratenon, nerves, and muscles on the hand/wrist and elbows, and result in disorders or diseases such as carpal tunnel syndrome, De Quervain’s diseases, vibration-induced white fingers, tendinitis, and lateral/medial epicondylitis [1,2,3,4,5]. In Member States of European Union, the prevalence of self-reported symptoms of WRULDs is about 14%–46%, and the corresponding cost is estimated at between 0.5% to 2% of the gross National product [6]. In the United States, the prevalence of WRULDs reported by interviewees is about 21.74%–33.90% [7]; together with low back pain, WRULDs costs about 45–54 billion annually (0.26% GNP in average) [8]. In Taiwan, the prevalence of WRULDs in different body parts is about 5.4% (elbows among female workers) to 14.4% (shoulders among female workers) [9]. Among that, the reported cases on musculoskeletal disorders was ranked first in the manufacturing industry (29.2%) in 2018. Furthermore, research has shown that workers usually perform their jobs, operate equipment and instruments, and use tools by hand every day; the frequent use of their upper extremities results in a higher risk for the upper extremities than for other body parts [10]. Therefore, preventing WRULDs has recently become an important issue.

The first step to prevent WRULDs is to recognize their risk factors. Previous studies have shown that biomechanical, psychosocial/organizational (e.g., job controls, job demands, coworkers and supervisor support, stress, etc.), and personal factors (e.g., gender, age, BMI, etc.) are associated with these WRULDs [1,2,3,4,5,7,10,11,12,13,14,15,16,17]. Gerr et al. (2014) [12,13] and Fan et al. (2009) [1] showed that workers with low social support, high job demand, and low job control increased the risk of hand/arm disorders and lateral epicondylitis [1,12]. The major biomechanical risk factors of WRULDs are over exertion, awkward posture, and high repetitiveness [2,3,5,8,10,11,13,14,15,16,18]. In addition, a study conducted by Keir et al. showed that forceful exertions are strongly associated with carpal tunnel syndrome, epicondylitis, and disorders of the hand wrist tendon [3]. Furthermore, study results also showed that workers exposed to forceful exertions (pinch force ≥ 8.9/9 N or grip force ≥ 44.1/45 N) were associated with increased risk of carpal tunnel syndrome or lateral epicondylitis [1,2]. Therefore, many checklists for assessing the risks of WRULDs have included section to evaluate these factors. For example, the ergonomic assessment worksheet (EAWS) has tables for assessing hand or finger force [19,20]. The key indicator method–manual handling operation (KIM–MHO) [21] and the occupational repetitive actions (OCRA) index [22] also include assessment tables for the force required to perform jobs. These rating systems to assess the forceful exertions correspond to the percentage of the maximum strength. However, it should be noticed that these checklists were developed from studies in which the subjects were Western; consequently, the criteria or thresholds in these checklists, such as hand or finger force in EAWS, were also Western based. There are, however, significant differences in physical difference (height, weight, BMI, etc.) and fitness and lifestyle between Western and Asian people, which might create differences in their force patterns [23,24,25,26,27,28,29,30,31]. Crosby et al. found that hobby demand could be used to predict the grip strength [25]. Frontera et al. also found that flexor and extensor muscles of study participants increased after 12 weeks of training [27]. Furthermore, the strength difference between the American young males and Chinese young males might be explained by the fact that the young Chinese students were encouraged to strive for academic achievements by parents and teacher, instead of physical performance [29]. Therefore, whether these criteria or thresholds are suitable for Asian people, such as the Taiwanese, still lacks investigation.

In addition, previous studies focused more on the measures and/or development of norm of grip strength since this value can be used to ensure the safety of manual work and help design working tools and products, even for clinical use [29,30,31,32,33,34,35,36,37,38,39,40,41,42,43,44,45,46]. The World Health Organization (WHO) recommends including force as a measure of the muscle’s function level for the International Classification of Functioning, Disability, and Health [47]. Few studies have focused on the pinch and press strength of hands [30,35,38,40,46].

One solution to this problem is to substitute the criteria or thresholds of hand force in the checklists. Therefore, the purpose of this study is to collect five types of the maximum hand strength among workers in the manufacturing industries in Taiwan according to the criteria in EAWS.

## 2. Materials and Methods

### 2.1. Study Participants

This study uses a cross-sectional design within a 3-month completion duration and a convenience sample. Based on the statistics from the Directorate-General of Budget, Accounting, and Statistics (Executive Yuan, R.O.C. (Taiwan)), there were around 3 million employees, aged from 15 to 64 years in the manufacturing industry in 2016. We used two steps to conduct the sampling. The first step was to systematically sample the 5 age strata with a 1 to 20,000 sampling rate due to time constrains. Next, we performed a sample size calculation based on the grip strength data from Liang’s study with statistic power = 0.8 and α = 0.05 [48]. The sample size was 20 for each age strata. To ensure the data followed normal distribution, we increased the sample size to at least 32 participants for each age strata. Therefore, there were 32, 48, 48, 38, and 32 participants in the age groups of 20–24, 25–34, 35–44, 45–54, and 55–64 years, respectively. In total, we recruited 198 subjects to participate in this study. All participants were operators and/or engineers working on the production line for at least 6 months in manufacturing industries in Central Taiwan. Exclusion criteria included (1) any musculoskeletal pain/symptoms in their upper extremities within 6 months; (2) any musculoskeletal disorders, e.g., carpal tunnel syndromes (CTS), trigger fingers, De Quervain’s disease, tendinitis, laterial/medial epicondylitis, etc., diagnosed by occupational physicians in the past; and (3) rheumatoid arthritis or heart diseases, which may affect the hand strength.

### 2.2. Measurement and Determination of Optimal Grip Span

Hand span was measured in both hands from the tip of the thumb to the tip of the small finger with the hand open as wide as possible using a Martin-type anthropometer. Based on the equations proposed by Ruiz et al. (2006) [49], the optimal grip span can be calculated, and then we can determine the best grip handle setting for the dynamometer.

A questionnaire was also used to collect and record some biographic, anthropometric, and job information. Biographic information included age and gender. Anthropometric information included height (cm), weight (kg), hand width (cm), and dominant hand. The dominant hand was determined by asking the question, “Which hand do you use for writing and eating?” Finally, we acquired their job information, including the company’s name, their department, job title, years in current position, etc.

All study participants read and signed the consent form before participating in the study. The consent form was approved by the Research Ethics Committee at China Medical University and Hospital, Taichung City, Taiwan (CMUH106-REC2-156).

### 2.3. Instrument and Testing Procedures

A standardized Jamar-configured hand grip dynamometer (Model: G200, Biometrics Ltd., Ynysddu, UK) was used to measure the grip strength. The rated load was between 0 to 90 Kg, with an accuracy less than 1% of the rated load. The grip span was used from 3.4 to 8.6 cm, with a 1.3 cm increment. All participants were set to the best grip span based on their hand span, as described previously.

A pinchmeter (Model: P200, Diameter: 45 × 6 mm, Biometrics Ltd., Ynysddu, UK) was used to measure the lateral pinch and palmar pinch strength. The rated load was between 0 to 22.5 kg, with an accuracy less than 0.6% of the rated load.

Both the grip dynamometer and pinchmeter were connected to a 16-channel BIOPCA MP 150 data acquisition and data analysis system (BIOPAC System, Inc., Goleta, CA, USA) via a general-purpose transducer amplifier (DA 100C, BIOPAC System Inc., Goleta, CA, USA). The sampling rate was 1000 Hz. Before the experiment started every day, the experimenters use the standard weights of 2 and 10 kg to calibrate the system.

A customized device was designed to measure the strength of the ball of the thumb and the thumb press. A polytetrafluoroethene-made square (8 cm × 6 cm × 3 cm) was attached to a load cell (LTZ-50KA, Kyowa Electronic Instruments Co., Ltd., Chofu, Japan). The load cell was screwed to a height-adjustable L-shape stainless steel stand, which was fixed to a table with C-shape clamps. The signals of the load cell were sent to a computer via a multifunction data acquisition device (USB-6002 multifunction I/O device, National Instruments Co., Austin, TX, USA). Customized software designed and written in LabView (National Instruments Co., Austin, TX, USA) was used to collect the data as a text file (.txt). The sampling rate was 1000 Hz.

**Figure 1:**
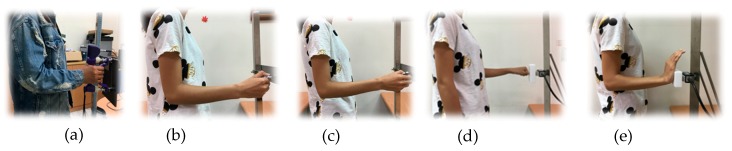
Posture and hand position of five hand exertions. (**a**) Grip; (**b**) Lateral pinch; (**c**) Palmar pinch; (**d**) Thumb press; (**e**) Ball of thumb.

The testing posture for grip and pinch strength followed the standardized positions recommended by the American Society of Hand Therapists (ASHT) and Mathiowetz et al. (1984), except in a standing position [50]. The participant stood with his/her shoulder adducted in a relaxed position, the elbow flexed at 90°, and the wrist in a neutral position. The forearm was in a neutral position for the grip and pinch tests and pronated 90° for the ball of thumb and thumb press (Figure 1). Grip and two pinch strength testing followed the instruction proposed by Mathiowetz et al. (1984) [50]. The thumb press and ball of thumb tests followed the recommendation of Shaub et al. (2015) [20].

Participants’ names, ages, genders, hand dominance, job information, and anthropometric data were recorded by a questionnaire. After explaining the instructions to the participant and the participant signed the informed consent, the participant started the experiment. Participants were asked to gradually exert force to their maximum voluntary contraction within 1–2 s and maintained that force for 5 s [51]. The mean of the strength data points in the middle of 3 s was calculated to represent the maximum strength for this specific trial. The strength of both the dominant and non-dominant hand for five different hand exertions was measured 3 times. The mean of the maximum strength of the three trials was calculated to represent the strength for a specific hand exertion. To ensure data reliability, we also calculated the coefficient of variation (CV) based on the data of the three trials. If the CV was greater than 10%, the participant was asked to perform the specific hand exertion again and the experimenter recalculated the CV. To avoid muscle fatigue, each participant only performed the specific hand exertion five times at most. In total, each participant performed hand exertions at least 30 times. There were at least 3 min rest periods in between the 2 trials to reduce the effect of fatigue [51]. The tests for the types of hand strength were randomized and counterbalanced. It took an hour to complete the experiment.

### 2.4. Statistical Analysis

There were 1980 strength data points collected (198 participants × 5 types of hand exertions × right/left hand). The mean values, standard deviation, and/or percent were used to present the demographic and strength data. A repeated measure ANOVA was used to determine the main effects and their interactions with age and the types of hand exertion. A Tukey test was performed for post hoc analysis. T-tests were performed to compare the hand strength of males vs. females (independent samples), and right vs. left hand (paired samples). A Pearson correlation coefficient was performed to determine the correlation between the independent variables. A *p*-value < 0.05 was used to determine statistical significance. All statistical analyses were performed using SPSS Chinese-version 22.0 (IBM Corporation, Armonk, NY, USA).

## 3. Results

### 3.1. Biographic Results of Study Participants

A total of 198 health persons were recruited in this study. Their demographic information and anthropometric information are shown in Table 1. There was no age difference between males and females (39.1 ± 12.8 vs. 39.1 ± 13.1 year, respectively (*p* = 0.974)). Regarding body mass index (BMI), there was a significant gender difference (*p* = 0.001). Although both males and females were in a normal weight category, the BMI of males reached the highest value in the normal weight category: 24.9 ± 3.8 kg/m^2^ for males and 23.2 ± 3.4 kg/m^2^ for females. For the hand span, males were significantly wider than females, 20.0 ± 1.9 vs. 17.8 ± 1.6 cm, respectively (*p* < 0.001). There were only 4 males (2.0%) and 20 females (10.1%) who needed to select the second handle as their optimum grip span. The other study participants used the third handle as their optimum grip span. There were only 12 participants with left-hand dominance (6.1%): Four among males and eight among females. Therefore, we only explored the right- and left-hand differences, instead of the differences between dominant and non–dominant hands.

After performing a K–S test to determine the data’s normality by gender, the original strength of the five types of hand exertion by gender did not follow a normal distribution. Therefore, we transformed the data by logarithm to base. After data transformation, the strength data followed a normal distribution. All statistical analyses used the transformation data.

### 3.2. Gender and Laterality Effects

Table 2 depicts that there were statistical differences among genders for all five types of hand exertions, which is not surprising (all *p* < 0.001). The strengths of the females for these five types of hand exertions were 52.0%–67.6% the strength of the males. Therefore, all the following comparisons are gender specific.

To compare the strength of the differences for both hands, the results revealed that the strength of the grip, lateral pinch, and palm pinch on the right hand were significantly stronger than the corresponding strengths on the left hand (*p* < 0.001). On the other hand, the strength of the ball of the thumb on the left hand was stronger than the corresponding strength on the right hand (paired-t_(98)_ = −2.255, *p* = 0.026)), but this was the only type of strength for which the left hand was stronger than the right hand among both genders. There was no difference in the strength of the thumb press for both hands in the male group (paired-t_(98)_ = 1.635, *p* = 0.105).

For females, the strength of the grip, thumb press, lateral pinch, and palm pinch were stronger on the right hand than the corresponding strength on the left hand (*p* < 0.001 ~ *p* = 0.002). There was no difference in the strength of the ball of the thumb for both hands (paired-t_(98)_ = 0.024, *p* = 0.981).

### 3.3. Effects of Types of Hand Exertions and Age on Strength

Since the results of Mauchly’s test of sphericity were significant (*p* < 0.001), the Greenhouse–Geusser method was applied to adjust for the determination of significant difference. The repeated-measures ANOVA results are shown in Table 3. Among the male participants, there was a main effect for the types of hand strength for the right-hand (F = 1078.381; df = 2.005, 188.461; *p* < 0.001) and the left-hand (F = 1094.502; df = 2.060, 193.595; *p* < 0.001). Post hoc test results revealed that grip strength was significantly greater than the other four types of hand exertions for both hands (*p* < 0.001). The strength of the ball of the thumb press was the second highest and was significantly greater than the thumb press strength and the strengths for the two types of pinch (*p* < 0.001). The lateral pinch strength of the right hand was 0.7 kgw greater than the palmar pinch strength (*p* < 0.001) but did not show a significant difference compared to the thumb press strength of the right hand. The palmar pinch strength of the right hand was the smallest hand strength among these five types of strength for the right hand, though there was no difference in the right hand thumb press strength. For the left hand, there were no differences in the hand strengths for the thumb press, lateral pinch, and palmar pinch. Meanwhile, there was no age effect on the five types of hand strength for both hands (F_(4,94)_ = 0.777, *p* = 0.543 for the left hand and F_(4,94)_ = 1.562, *p* = 0.191 for the left hand). On the other hand, there was an interaction between the types of hand exertions and age for the right-hand (F = 2.795; df = 8.02, 188.461; *p* = 0.006) and the left-hand (F = 3.041; df = 8.238, 193.595; *p* = 0.003).

For females, there was a main effect for the types of hand strength for the right-hand (F = 830.198; df = 2.120, 199.298; *p* < 0.001) and the left-hand (F = 923.448; df = 2.045, 192.266; *p* < 0.001). Post hoc test results unsurprisingly revealed that the grip strength was the highest hand strength among these five types of hand exertions for both hands (*p* < 0.001). The results were the same for the males: The strength of the ball of the thumb press was the second highest strength compared to the thumb press strength, and the lateral and palmar pinch strength for both hands among female participants (*p* < 0.001). However, there was no significant difference in the strength between the thumb press, lateral pinch, and palmar pinch for both hands among females. Furthermore, there were no significant difference for the age for both hands (F_(4,94)_ = 1.333, *p* = 0.264 for the right hand and F_(4,94)_ = 0.503, *p* = 0.734 for the left hand). In addition, there was no significant interactions between the types of hand exertions and age for both hands.

#### 3.3.1. Grip Strength

It was unsurprising that the grip strength of females was 44.9% to 61.6% weaker than the strength of the males in all age groups (*p* < 0.001). The grip strength of the males reached its maximum (46.7 ± 9.8 kgw) among the age group of 25–34 years and decreased as the age increased on the right hand (Table 4). The grip strength at the age of 55–64 years was significantly lower than the strength at 25–34 and 35–44 years. In contrast, this pattern did not occur for females, though the maximum grip strength was found for the age group of 25–34 years (Table 5). The minimum grip strength was at 35–44 years. There was no significant difference in the grip strength among the different age groups. For the left hands of male participants, the grip strength again reached its maximum (45.1 ± 7.8 kgw) at the age of 25–34 years and decreased as the age increased. Again, there was no such trend observed for the left hand among the female participants.

#### 3.3.2. Ball of the Thumb Press

The ball of the thumb press strength for females was significantly lower than the strength of males across the five age groups (59.3%–74.2%, *p* < 0.001). The strengths of the ball of the thumb reached their maximum at the age of 45–54 years for both hands among males and females. There was a significant difference in the strength of age effects for male participants (F_(4,94)_ = 2.475, *p* = 0.05). Among males, the strength of the ball of the thumb press at the age of 45–54 years was 3.33 kgw higher than the strength at the age of 35–44 years (*p* = 0.04). There were no differences in the ball of the thumb press among other age groups. Furthermore, the strength of the ball of the thumb on the left hand was 0.28 kgw higher than the right hand (F_(4,94)_ = 5.315, *p* = 0.023), which was the only type of hand exertion for which the left-hand strength was greater than the right-hand strength. There was no significant interaction between hands and age groups.

For female participants, there were no main effects between hands and ages on the strength of the ball of the thumb press (F_(1,94)_ = 0.281, *p* = 0.597 for hands and F_(4,94)_ = 0.854, *p* = 0.494 for ages). The maximum strength of the ball of the thumb was 8.1 ± 2.8 kgw at the age of 45–54 years and the minimum strength was 6.8 ± 2.0 kgw at the age of 55–64 years on their right hands. No interaction between ages and hands was observed (F_(4,94)_ = 0.621, *p* = 0.649).

#### 3.3.3. Thumb Press

The strength of thumb press for females was significantly lower (50.6%–70.2%) than the strength for males across the five age groups. For male participants, hands played a marginal role in the thumb press strength (F_(1,94)_ = 3.516, *p* = 0.064). There were no main effects resulting from ages (F_(4,94)_ = 0.974, *p* = 0.426) nor were there any interaction between age and hand (F_(4,94)_ = 1.258, *p* = 0.292) for the thumb press. The maximum strengths of the thumb press were 9.7 ± 3.7 kgw and 9.7 ± 4.2 kgw at the age of 45–54 years on the right- and left-hand. The minimum strength was 8.2 ± 2.2 kgw at the age of 35–44 years on the right-hand and was 8.0 ± 2.5 kgw at the age of 20–24 years on the left-hand.

For females, the strength of the thumb press on the right–hand was significantly greater than the strength on the left-hand (F_(1,94)_ = 14.895, *p* < 0.001), especially at the age of 20–24 and 25–34 years. There was no main effect of age (F_(4,94)_ = 1.635, *p* = 0.172) and age and hand interactions (F_(4,94)_ = 1.248, *p* = 0.282) on the thumb press. The maximum strength of the thumb press on the right hand was 5.9 ± 1.4 kgw at 20–24 years and decreased as the age increased. For the left hand, the maximum strength was 5.5 ± 1.7 kgw at 25–34 years.

#### 3.3.4. Lateral Pinch

For the lateral pinch, females’ strengths were 2.3–3.8 kgw (57.8%–69.7%) less than the males’ strengths among all age groups (*p* < 0.001). For male participants, the main effects of hand and age on the strengths of lateral pinch were significant (F_(1,94)_ = 24.38, *p* < 0.001 for hand; F_(4,94)_ = 3.062, *p* = 0.02 for age). The lateral pinch strength on the right hands was 0.53 ± 0.11 kgw higher than the strength on the left hand. The lateral pinch strength reached its maximum among the age group of 25–34 years; this strength was 1.52 ± 0.47 kgw higher than the minimum strength at the age of 20–24 years. Although the strength of the lateral pinch decreased as the age increased, there was not a significant difference. There was no significant difference in the interactions between hand and age (F_(4,94)_ = 0.855, *p* = 0.494).

For female participants, there was a significant main effect of the hand on the strength of the lateral pinch (F_(1,94)_ = 26.137, *p* < 0.001). The strength of the lateral pinch on the right hands was significantly higher than the strength of the left hand for those aged 20–54 years, except at the age of 55–64 years. The mean difference of the lateral pinch strength on the right- and left-hand was 0.38 ± 0.07 kgw. The maximum and minimum strength of the lateral pinch on the right hand was 5.7 ± 1.1 kgw at 45–54 years and 5.1 ± 1.2 kgw at 20–24 years. Furthermore, the maximum and minimum strength of the lateral pinch on the left hand was 5.3 ± 1.4 kgw at 55–64 years and 4.7 ± 1.0 kgw at 20–24 years. There was no main effect of the age (F_(4,94)_ = 0.723, *p* = 0.578) nor an interaction of the age and hand (F_(4,94)_ = 1.676, *p* = 0.162) on the lateral pinch for females.

#### 3.3.5. Palmar Pinch

The strength of the palmar pinch for females was 60.8%–74.2% lower (1.7–3.1 kgw) than the strength of males among all age groups (*p* < 0.001 to *p* = 0.003). For male participants, there were significant main effects of hand and age on the palmar pinch (F_(1,94)_ = 25.172, *p* < 0.001 for hand; F_(4,94)_ = 2.708, *p* = 0.035 for age). On average, the strength of the palmar pinch for the right hands was 0.57 ± 0.11 kgw higher than the strength for the left hands. The maximum strength of the palmar pinch on the right hands was 8.4 ± 1.8 and 8.4 ± 1.6 kgw at the age of 25–34 years and 45–54 years. For the left hands, the maximum strength was 8.1 ± 1.7 kgw at the age of 25–34 years. The minimum strength of the palmar pinch for the right- and left-hand was 7.2 ± 1.3 kgw and 6.6 ± 1.6 at the age of 55–64 years, respectively.

For females, a main effect of the hand on the strength of the palmar pinch revealed significant differences (F_(1,94)_ = 10.543, *p* = 0.002). The strength of the palmar pinch on the right hand was 0.30 ± 0.09 kgw higher than the strength on the left hands. There were no significant differences in the palmar pinch across all age groups. The maximum strength of the palmar pinch for the right- and left-hand was 5.6 ± 1.0 and 5.3 ± 0.9 kgw at the age of 25–34 years, respectively. The minimum strength was 5.1 ± 1.4 kgw at 34–44 years for the right hand and 4.8 ± 1.1 kgw at 45–54 years for the left hand. However, the difference in the strength was relatively small among these five age groups. In addition, there was no significant interactions between hand and age on the palmar pinch strength.

### 3.4. Correlation Coefficients

A bivariate correlation matrix of the strength data for the right- and left-hand, as well as biographic and anthropometric data, are shown in Table 6. The results of the strength of the two hands are highly correlated with the coefficient from 0.867 to 0.964, regardless of the type of hand exertion. The highest correlation coefficient occurred for the ball of the thumb press (*p* < 0.001), and the lowest correlation occurred for the palmar strength (r = 0.755–0.823, *p* < 0.001). On the other hand, the grip strength for both hands was moderately correlated with the strength of the ball of the thumb and the thumb press (r = 0.452–0.654, *p* < 0.001). Both types of pinch strength on the right- and left-hands were also highly correlated (r = 0.791–0.910, *p* < 0.001). In addition, the pinch strengths and press strengths for both hands were moderately correlated (r = 0.419–0.610, *p* < 0.001). The strength of the ball of the thumb on both hands was highly correlated with the strength of thumb press (r = 0.823–0.964, *p* < 0.001), but were moderately correlated with the grip and pinch strength. Age was weakly correlated with BMI (r = 0.166, *p* < 0.05) and negatively correlated with height (r = −0.216, *p* < 0.05). Gender was highly correlated with the grip strength and lateral pinch strength for both hands, but was moderately correlated with the palmar strength, ball of the thumb press, and the thumb press. Height was also highly correlated with the grip strength (r = 0.754 for right hand and r = 0.731 for left hand, *p* < 0.001), but was moderately correlated with all types of press and pinch strengths. On the other hand, all five types of hand strengths were moderately correlated with weight.

## 4. Discussion

The test of grip strength followed the testing procedures recommended by ASHT, except the testing was undertaken from standing positions in this study. The represented maximum grip strength was the mean of the results from the three trials. Mathiowetz et al. (1984) recommends that the highest precision grip strength should use a Jamar Dynamometer and the highest reliability should be based on the mean of three trials, rather than the results from one trial or two trials [50]. In contrast, Nilsen et al. (2012) recommends that the results of one trial are as reliable as the results of three trials, and study participants may feel muscle fatigue and pain after multiple trials [43]. Many grip strengths from the first trial were either too high (jerk force) or too low in our study and resulted in lower reliability. A possible reason for this result is that the study participants were not familiar with the use of the instrument, although the experimenters gave an opportunity for practice. Therefore, we still recommend that hand strength results should be tested three times.

Among the five types of hand strength, the order of right-hand strength from high to low are grips (31.9 kgw), ball of thumb (9.2 kgw), lateral pinch (7.1 kgw), thumb press (7.0 kgw), and palmar pinch (6.6 kgw). It is not surprising that the muscle groups used for grip strength are all muscles of the upper limbs. Further, the participants used more muscle groups of the upper limbs when performing a ball of thumb, and this strength was ranked second. The muscle groups used to perform the pinch were relatively fewer than those used for the ball of thumb, and the strength was smaller. Moreover, the lateral pinch strength was greater than the palmar pinch strength, which agrees with the results from previous studies [35,40,42].

Grip strength was the most important factor in predicting hand functionality and other factors, such as nutrition effects [10,23,52,53,54,55,56,57]. Bohannon et al. (2015) mentioned that the grip strength is an indicator of the muscular conditions of the upper limbs and can be used for clinical evaluation [58]. In this study, the maximum difference occurred between the palmar pinch strength and the grip strength on the left hand. The palmar pinch strength was only 20.5% that of the grip strength on the left hand. The minimum difference was found between the ball of thumb and grip strength of both hands. The ball of thumb strength on the left hand was about 30.8% of the grip strength of the left hand, and the ball of thumb strength of the right hand was about 28.8% of the grip strength of the right hand.

The study results revealed that the five types of hand strength for both hands were significantly higher for males than for females. The present study results revealed similar conclusions: The female grip strength was about 52%–55% of the grip strength of males in Taiwan [29,59]. The possible reasons for these results are the difference in muscle fiber specificity, the ratio of the composition of fat, bone, and muscle, and physical training. In addition, males are taller and heavier than females, which results in differences in the muscle mass between genders.

Compared with the results from international studies, the grip strength of both genders in Taiwan was significantly smaller (59% to 64%) than the strength in U.S. and European countries (Table 7). In terms of the gender effects on pinch strength, the lateral pinch strength for females was significantly lower than the strength of males for the right and left hands in this study (62.1% and 61%, respectively). This result is slightly smaller than the results from previous studies in the U.S. and European countries, which showed that the lateral pinch strength for females was 66%–68% that of males (Table 8) [38,40,46]. In addition, the strength of the palmar pinch for females was also significantly lower than the strength of males on the right and left hand in this study (66.3% and 67.6%, respectively). This result is nearly identical to the study results by Mathiowetz et al. (1985) and Harth and Vetter (1994), which showed that palmar pinch strength for females was 69.7% and 67.9% that of males [38,40]. For the two types of press, the female strengths were also 60.0%–66.4% of the strength of males. However, studies on the two types of hand exertions to make any comparisons to international studies are scarce. In general, race, which accounts for body shape and muscle mass, also plays an important role in the effect of hand strength. In addition, studies also show that recreation and regular exercise had a positive association with grip strength. In future studies, we should record information relevant to these two factors. Besides race, these two reasons can also be used to explain the differences between the grip strength in Taiwan and in U.S./European countries, especially for females (Table 7). In Taiwan, females generally do not engage in regular outdoor exercise or recreations since childhood. In contrast, most females in U.S./European countries engage in routine exercise or recreation. This cultural difference also plays an important role in the differences of muscle strength and agrees with previous studies [29,30,31,33,36,40]. Therefore, applying assessment tools that were mainly designed by scholars in U.S. and European countries to evaluate the risks of musculoskeletal disorders in Taiwan should be more conservative; revising the numbers and validating the results are recommended.

Another factor affecting grip strength is age [52]. As age increases, the deterioration of the musculoskeletal system, decreases in muscle mass, decreases in the elasticity of soft tissue, and restrictions in the range of motion of joints, motor-neuron abnormalities, and decreases in daily activities contribute to a decrease in grip strength [28]. In this study, the grip strength reached its peak at the age of 25–39 years for males and significantly declined after 40 to 45 years. This result is inconsistent with the study results conducted by Su et al. and Mathiowetz et al., which showed that male grip strength was highest around 20–39 years of age [29,40]. The peak grip strength for females is identical to the results of Mathiowetz’s study that showed female grip strength to reach its peak at 30–34 years [40]. However, these results conflicted with the results from two other studies in Taiwan, which showed that the maximum grip strength occurs from 40 to 54 years [29,30]. The possible reasons for this disagreement are the body position (sitting vs. standing) and occupations. In this study, the study participants were mainly from the manufacturing industry. On the other hand, the participants recruited by Su et al. and Wu et al. were taken from across all occupations, including housewives and retired senior citizens with convenient samplings in Taiwan. Similar to earlier descriptions, recreation and regular exercise had a positive association with grip strength. In comparison with the Hong Kong study, the grip strengths in the present study were significantly higher than those in the previous study across all age strata [31]. These results, however, contradict Korean studies. The grip strengths of males tested by Han et al. were higher than the strengths in this study, except for elderly participants [35]. On the other hand, the grip strengths were similar between the studies conducted by Kim et al. and the present study, except for participants above 45 years of age [39]. Compared to studies in the U.S., the grip strengths of males were also higher than the strengths of males in Taiwan across all age groups [33,36,40,45]. However, the differences in grip strength were small (about 15%) at age 25–44 years. Young (20–24) and older (45–65) male adults in the U.S. had a significantly higher grip strength than male adults from similar age groups in Taiwan. Nonetheless, one study recently published by Wang et al. (2018) showed that the grip strength of male adults in the U.S. was similar to the strength of males in Taiwan at the age of 25–44 years [45]. Comparing the strength of Taiwanese male adults to those in European countries, the grip strength in Taiwan males was 72%–88% smaller than the strength in Norway, Finland, Germany, and Switzerland [34,37,38,43,44,46]. On the other hand, the grip strength of males in this study was 1.2–1.4 times higher than the grip strength in Nigeria [32]. For females in Taiwan, the grip strength in this study was similar to the results conducted by Wu et al. (2009) [30], but significantly lower than the study results conducted by Su et al. (1994) [29] (Table 8). In comparison with countries in Asia, the grip strengths of females in this study was 3%–6% higher than those of females in Hong Kong, except for the age group of 35–44 years [31]. The female grip strength in Korea was 1.25 to 1.66 times higher than the strength in this study [35,39]. Compared to studies in the U.S., the grip strength of females was also higher than the strength of females in this study across all age groups [33,36,40,45]. Comparing the strength of Taiwanese female adults to those in European countries, the grip strength in this study was only 59%–70% of the female strength in Norway, Finland, Germany, and Switzerland [34,38,43,44,46].

In comparison with studies in other countries, the strength of the lateral pinch in Taiwan was significantly less (Table 8). For Taiwanese males, the lateral pinch strength was 1.5 to 2 times higher than Wu’s study in 2009 [30]. Compared with the study in Korea, the lateral pinch strength of males was about 1.2 times higher than the strength in this study [17,18]. It is unsurprising that the lateral pinch strength in this study was significantly lower than that in the U.S. and European countries, even compared to other countries in Asia (Table 8). In comparison with Han’s study in Korea, the lateral pinch strength of females was about 1.3 times greater than the strength in this study [17]. The peak strength of the lateral pinch for female participants occurred at 30–44 years in Korea, the U.S., and European countries. However, the peak lateral pinch strength was found at of 45–54 years in this study.

The strength of the male palmar pinch on the right hand declined 14.3% from the ages of 45–54 years to 55–64 years in this study; these results agree with the Korean results, which showed that the strength declines less than 20% between 20 to 59 years and experiences a 30.3% reduction after 60 years of age among Koreans [35] (Table 9). Additionally, the palmar pinch strength of males in this study was 78%–84% of the male strength in Korea at 30–59 years of age [35]. The strength at the age of 20–29 and greater than 60 years was similar among these two studies. The palmar pinch strength of males in U.S. was 1.3–1.5 times higher than the strength in this study [40]. However, the results in the present study disagree with those of a study conducted in Germany, which showed that there is no strength difference between the ages of 20 and 59 years, but the palmar pinch strength increased by 12.8% after 65 years of age [38]. In general, the peak palmar pinch strength of males occurs at the age of 25–39 years in Korea, the U.S., and Taiwan. On the other hand, the peak palmar pinch strength occurs at 60–69 years in Germany.

For females, changes in palmar pinch strength are less than 10% before 50 years of age. In Korea, palmar strength declined by 14.9% at the age of 60 years and strength declined up to 20.9% above 70 years old when comparing palmar strength to the age group of 30–39 years. The palmar pinch strength in this study was 76%–91% of the strength in Korea. In comparison with Germany and the U.S., the palmar pinch strength in this study was 63.6%–77.6% the strength of these previous studies [38,40]. The peak palmar pinch strength of females occurred at 20–39 years of age. It is interesting that the strength of the palmar pinch for Taiwanese males was the same as the strength of females in Germany and the U.S. When making a comparison to the palmar pinch strength in Norway, the maximum strengths were smaller than the strengths in our study [43]. This difference is because the methods of hand exertions between these two studies were different. The researchers measured the palmar pinch by asking the participants to apply forces using their thumb and index finger (a two-finger palmar pinch). On the other hand, we asked the participants to exert force using their thumb, index finger, and middle finger (a three-finger palmar pinch). It is reasonable that a three-finger palmar pinch strength test would provide greater results than a two-finger palmar strength test.

Previous epidemiological studies determined the forceful exertions by the values of 9 N (or 0.9 kgw) for pinch strength and 44 N (4.5 kgw) for grip strength when performing the tasks. They found that forceful repetition rate were associated with the increase of carpal tunnel syndrome and/or lateral epicondylitis [1,2]. The grip force was about 19.1% (4.5 kgw/23.6 kgw) of the females and 11.7% (4.5 kgw/38.4 kgw) of the males in the U.S. In addition, the pinch force was about 13.8% (0.9 kgw/7 kgw) and 8.6% (0.9 kgw/10.5 kgw) for females and males, respectively. However, it was about 22.1% (4.5 kgw/20.3 kgw) of the female grip strength and 12.7% (4.5 kgw/35.5 kgw) of the male grip strength, and 17.6% (0.9 kgw/5.1 kgw) of the female lateral pinch strength and 11.7% (0.9 kgw/7.7 kgw) of the male lateral pinch strength in this study. Clearly, the cut-off values for grip and pinch forces are relatively greater for Taiwanese workers. Again, future laboratory and epidemiological studies are needed to revise those criteria or thresholds of hand force in the checklists.

There are some strengths in this study. First, we measured the maximum hand strength three times for five types of hand exertions, which can increase reliability and minimize intra-variability. Second, all study participants recruited were from the production department in the manufacturing industry, so these results can be directly applied to design tools and jobs on the assembly line. On the other hand, there are some limitations. First, this was a cross-sectional study and we can only use those factors to determine the association to the strength, instead of investigating the causation. When recruiting the study participants, we did not consider the sub-categories within the manufacturing industry. Different types of jobs and hand exertions when performing work might affect hand strength. We also did not collect some demographic information, such as leisure activities and types of exercise. In addition, the study participants were from the manufacturing industry in this study, so the participants’ hand strengths cannot represent the entire labor force in Taiwan. One ongoing project by the authors focuses on a study population in other industries.

## 5. Conclusions

This study is the first to investigate the norms of strength among five different types of hand exertions among healthy manufacturing workers in Taiwan. The strength of females for these five types of hand exertions was 52.0%–67.6% of the strength of males. For both genders, there was a main effect of the types of hand strength for the right-hand and left-hand. In general, hand strength in the U.S. and EU countries was 1.2 to 1.7 times greater than the strength among the three types of hand exertions in this study. Therefore, those cut-off values of forceful exertions used to evaluate the musculoskeletal burdens of upper extremities in the manufacturing industry may need to be justified. Furthermore, these results can be used to determine the weight for tool and job designing, as well as job modifications.

## Figures and Tables

**Table 1:** Demographic information of the participants (*n* = 198).

	Male (*n* = 99)	Female (*n* = 99)	*p*-Value ^c^	Total (*n* = 198)
	Mean ± SD n (%)	Mean ± SD n (%)		Mean ± SD n (%)
Age (year) ^a^	39.1 ± 12.8	39.1 ± 13.1	0.974	39.1 ± 12.9
Height (cm) ^a^	171.9 ± 5.9	159.5 ± 5.0	<0.001 **	165.7 ± 8.3
Weight (Kg) ^a^	73.7 ± 12.8	59.0 ± 9.3	<0.001 **	66.3 ± 13.4
BMI (Kg/m^2^) ^a^	24.9 ± 3.8	23.2 ± 3.4	0.001 *	24.0 ± 3.7
Hand span (cm) ^a^	20.0 ± 1.9	17.8 ± 1.6	<0.001 **	18.9 ± 2.0
Handle (count) ^b^				
2	4 (2.0%)	20 (10.1%)		2 (12.1%)
3	95 (48.0%)	79 (39.9%)		174 (87.9%)
Dominance (count) ^b^				
Right	95 (48.0%)	91 (46.0%)		186 (93.9%)
Left	4 (2.0%)	8 (4.0%)		12 (6.1%)

Note: ^a^: t-Test; ^b^: Handle 2 was the 2nd handle of dynamometer (4.7 cm); Handle 3 was the 3rd handle of dynamometer (6.0 cm); ^c^: * *p* < 0.05; ** *p* < 0.001.

**Table 2:** Strength data by type of hand exertion, gender, and right- and left-hand (Unit: kgw).

Type of Hand Exertion	Hand	Males (*n* = 99)				Females (*n* = 99)				Female/Male Ratio
		Mean ± SD	Max.	Min	*p*-Value ^2^	Mean ± SD	Max.	Min	*p*-Value ^2^	
Grip	R	41.9 ± 9.4	63.9	18.1	*p* < 0.001 *	21.8 ± 5.1	32.3	9.9	*p* < 0.001 *	52.0% ^1^
	L	39.7 ± 8.4	59.9	22.5		20.8 ± 5.0	35.8	10.2		52.4% ^1^
Ball of Thumb	R	11.0 ± 3.9	24.0	4.5	*p* = 0.026 *	7.3 ± 2.3	15.0	3.4	*p* = 0.981	66.4% ^1^
	L	11.3 ± 4.1	25.3	4.7		7.2 ± 2.1	13.6	3.5		63.7% ^1^
Thumb Press	R	8.6 ± 2.8	17.1	4.6	*p* = 0.105	5.3 ± 1.8	10.3	2.1	*p* = 0.002 *	61.6% ^1^
	L	8.5 ± 2.8	18.3	4.0		5.1 ± 1.6	10.3	2.3		60.0% ^1^
Lateral Pinch	R	8.7 ± 1.6	12.5	5.4	*p* < 0.001 *	5.4 ± 1.2	9	2.6	*p* < 0.001 *	62.1% ^1^
	L	8.2 ± 1.7	12.9	3.7		5.0 ± 1.2	8.9	2.4		61.0% ^1^
Palmar Pinch	R	8.0 ± 1.7	13.3	4.5	*p* < 0.001 *	5.3 ± 1.3	9.6	2.8	*p* = 0.001 *	66.3% ^1^
	L	7.4 ± 1.7	12.4	3.1		5.0 ± 1.1	8.8	2.5		67.6% ^1^

^1^ Student’s t test and *p* < 0.001; ^2^ Pair-t test. *: *p* < 0.05.

**Table 3:** Repeated-measures ANOVA of the strength for the effects of the types of hand exertions and age group.

Gender	Hand	Source	Sum of Squares	Degree of Freedom	Mean Square	F	*p*-Value
Males	Right	Within Subject					
		Type of Exertion	34.945	2.005	17.430	1078.381	<0.001 **
		Type of Exertion × Age	0.362	8.020	0.045	2.795	0.006 *
		Error	3.046	188.461	0.016		
		Between subject					
		Age	0.098	4	0.024	0.777	0.543
		Error	2.959	94	0.031		
	Left	Within Subject					
		Type of Exertion	34.865	2.060	16.929	1094.502	<0.001 **
		Type of Exertion × Age	0.387	8.238	0.047	3.041	0.003 *
		Error	2.994	193.595	0.015		
		Between subject					
		Age	0.198	4	0.050	1.562	0.191
		Error	2.981	94	0.032		
Females	Right	Within Subject					
		Type of Exertion	27.358	2.120	12.903	830.198	<0.001 **
		Type of Exertion × Age	0.195	8.481	0.023	1.480	0.162
		Error	3.098	199.298	0.016	-	-
		Between subject					
		Age	0.196	4	0.049	1.333	0.264
		Error	3.461	94	0.037		
	Left	Within Subject					
		Type of Exertion	27.479	2.045	13.435	923.448	<0.001 **
		Type of Exertion × Age	0.178	8.182	0.022	1.496	0.159
		Error	2.797	192.266	0.015		
		Between subject					
		Age	0.07	4	0.017	0.503	0.734
		Error	3.269	94	0.035		

Note: * *p* < 0.05; ** *p* < 0.001

**Table 4:** Five types of hand strength on both hands by age strata for male participants (*n* = 99, unit: kgw).

Age Strata/Hand	Grip		Ball of Thumb		Thumb Press		Lateral Pinch		Palmer Pinch	
	Mean ± SD	Range	Mean ± SD	Range	Mean ± SD	Range	Mean ± SD	Range	Mean ± SD	Range
20–24										
Right	40.8 ± 9.6	26.0–55.1	12.0 ± 3.6	6.0–17.7	8.4 ± 2.9	5.2–13.7	7.7 ± 1.2	6.0–11.1	7.9 ± 2.2	5.0–13.3
Left	35.3 ± 7.3	24.4–45.2	11.9 ± 3.7	5.8–17.7	8.0 ± 2.5	5.0–12.7	7.5 ± 1.1	5.8–9.6	7.2 ± 1.8	5.2–12.4
25–34										
Right	46.7 ± 9.8	18.1–63.0	10.3 ± 2.8	5.2–15.4	8.3 ± 2.6	5.2–14.9	9.3 ± 1.3	5.6–11.5	8.4 ± 1.8	4.5–12.7
Left	45.1 ± 7.8	26.7–57.3	10.5 ± 3.1	4.7–18.7	8.3 ± 2.7	4.5–15.4	9.0 ± 1.6	6.4–12.8	8.1 ± 1.7	5.6–12.4
35–44										
Right	45.2 ± 8.9	22.4–63.9	9.7 ± 2.3	6.0–13.9	8.2 ± 2.2	5.0–12.4	8.9 ± 1.5	3.6–12.3	7.7 ± 1.2	6.2–10.3
Left	42.9 ± 8.2	23.3–59.9	9.9 ± 2.2	5.8–13.6	8.1 ± 2.1	5.0–13.1	8.1 ± 1.5	5.9–10.6	6.9 ± 1.1	5.4–9.9
45–54										
Right	38.1 ± 8.2	22.9–55.7	12.8 ± 5.9	4.5–24.0	9.7 ± 3.7	4.6–17.1	8.9 ± 1.9	5.4–12.5	8.4 ± 1.6	6.0–11.6
Left	37.5 ± 8.0	22.5–51.0	13.5 ± 6.2	5.0–25.3	9.7 ± 4.5	4.0–18.3	8.3 ± 2.1	4.5–12.9	7.9 ± 2.0	4.5–11.9
55–64										
Right	35.5 ± 4.3	29.5–42.9	10.9 ± 3.6	7.7–19.2	8.7 ± 2.9	4.6–15.0	8.3 ± 1.5	5.9–11.1	7.2 ± 1.3	5.7–10.0
Left	33.3 ± 3.2	30.3–39.0	11.4 ± 3.9	7.7–18.7	8.2 ± 2.1	5.1–11.8	7.6 ± 1.6	3.7–10.7	6.6 ± 1.6	3.1–9.8

**Table 5:** Five types of hand exertions of strength on both hands by age strata for female participants (*n* = 99, unit: kgw).

Age Strata/Hand	Grip		Ball of Thumb		Thumb Press		Lateral Pinch		Palmar Pinch	
	Mean ± SD	Range	Mean ± SD	Range	Mean ± SD	Range	Mean ± SD	Range	Mean ± SD	Range
20–24										
Right	22.6 ± 4.9	15.2–32.3	7.0 ± 1.5	4.0–9.6	5.9 ± 1.4	3.2–8.4	5.1 ± 1.2	2.6–7.4	5.2 ± 1.2	3.1–8.2
Left	20.2 ± 4.6	13.4–27.9	6.9 ± 1.6	3.9–10.4	5.4 ±1.5	2.8–8.2	4.7 ± 1.0	2.8–6.8	4.9 ± 0.9	3.5–6.4
25–34										
Right	23.4 ± 5.3	14.8–32.2	7.2 ± 2.6	4.3–15.0	5.7 ± 1.7	3.8–10.2	5.6 ± 1.0	2.9–6.9	5.6 ± 1.0	4.0–7.4
Left	21.0 ± 4.7	14.4–29.5	7.1 ± 2.5	4.1–13.6	5.5 ± 1.7	3.9–10.3	5.2 ± 1.1	2.4–7.7	5.3 ± 0.9	3.9–6.8
35–44										
Right	20.3 ± 5.4	9.9–29.6	7.2 ± 2.1	4.4–12.4	5.2 ± 2.0	2.1–9.8	5.3 ± 1.3	3.4–9.0	5.1 ± 1.4	2.9–9.2
Left	19.9 ± 5.1	10.2–28.4	7.2 ± 2.1	4.4–12.6	5.0 ± 1.8	2.3–9.0	5.0 ± 1.3	3.0–8.2	4.9 ± 1.2	2.7–8.1
45–54										
Right	22.4 ± 4.3	17.0–32.2	8.1 ± 2.8	3.6–13.8	5.4 ± 2.1	2.5–10.3	5.7 ± 1.1	4.2–7.6	5.3 ± 1.2	3.7–8.5
Left	22.1 ± 5.0	14.9–34.2	8.0 ± 2.4	3.5–11.8	5.0 ± 1.6	3.0–9.1	5.0 ± 1.0	3.6–7.5	4.8 ± 1.1	3.4–7.8
55–64										
Right	20.4 ± 4.8	10.6–29.7	6.8 ± 2.0	3.4–10.0	4.4 ± 1.3	2.6–6.6	5.4 ± 1.2	3.6–8.3	5.2 ± 1.6	2.8–9.6
Left	20.5 ± 5.8	11.6–35.8	7.0 ± 1.9	3.9–11.1	4.4 ±1.3	2.5–6.9	5.3 ± 1.2	3.7–8.9	4.9 ± 1.5	2.5–8.8

**Table 6:** Persons correlation coefficients between five types of hand strengths, demographic information, and anthropometric measures.

Variables	1	2	3	4	5	6	7	8	9	10	11	12	13	14	15	16
1. R-Grip	1	-	-	-	-	-	-	-	-	-	-	-	-	-	-	-
2. L-Grip	0.960 **	1														
3. R-Ball of Thumb	0.465 **	0.461 **	1													
4. L-Ball of Thumb	0.452 **	0.467 **	0.964 **	1												
5. R-Thumb Press	0.618 **	0.616 **	0.845 **	0.832 **	1											
6. L-Thumb Press	0.645 **	0.654 **	0.823 **	0.824 **	0.961 **	1										
7. R-Lateral Pinch	0.822 **	0.823 **	0.429 **	0.425 **	0.575 **	0.594 **	1									
8. L-Lateral Pinch	0.804 **	0.820 **	0.419 **	0.434 **	0.580 **	0.605 **	0.910 **	1								
9. R-Palmar pinch	0.782 **	0.758 **	0.443 **	0.444 **	0.575 **	0.597 **	0.827 **	0.791 **	1							
10. L-Palmer Pinch	0.755 **	0.758 **	0.432 **	0.431 **	0.589 **	0.610 **	0.814 **	0.893 **	0.867 **	1						
11. Age	−0.133	−0.065	0.010	0.042	−0.039	−0.030	0.036	0.013	−0.058	−0.071	1					
12. Gender	0.801 **	0.809 **	0.508 **	0.530 **	0.571 **	0.593 **	0.766 **	0.741 **	0.672 **	0.649 **	0.002	1				
13. BMI	0.276 **	0.318 **	0.274 **	0.24 *	0.191 *	0.207 *	0295 **	0.286 **	0.207 *	0.245 **	0.166 *	0.235 *	1			
14. Hand Width	0.462 **	0.472 **	0.360 **	0.362 **	0.365 **	0.357 **	0.387 **	0.348 **	0.366 **	0.344 **	0.091	0.519 **	0.286 **	1		
15. Height	0.754 **	0.731 **	0.475 **	0.472 **	0.553 **	0.567 **	0.682 **	0.655 **	0.616 **	0.588 **	–0.216 *	0.753 **	0.190 *	0.435 **	1	
16. Weight	0.580 **	0.603 **	0.445 **	0.416 **	0.412 **	0.431 **	0.554 **	0.534 **	0.455 **	0.647 **	0.014	0.550 **	0.871 **	0.438 **	0.639 **	1

Note: *: *p* < 0.05; **: *p* < 0.001.

**Table 7:** Comparison of grip strength among countries (unit: kgw).

Studies	Sex ^a^	Age Strata ^b^																	
		20–24	(20–29)	25–29	(25–34)	30–34	(30–39)	35–39	(35–44)	40–44	(40–49)	45–49	(45–54)	50–54	(50–59)	55–59	(55–64)	60–64	(60–69)
This study	M	40.8	–	–	**46.7**	–	–	–	45.2	–	–	–	38.1	–	–	–	35.5	–	–
Taiwan	F	22.6	–	–	**23.4**	–	–	–	20.3	–	–	–	22.4	–	–	–	20.4	–	–
Wu et al.	M	38.5	–	**40.0**	–	39.0	–	38.6	–	36.6	–	36.8	–	36.1	–	35.3	–	28.3	–
Taiwan (2009) [30]	F	21.9	–	22.3	–	23.6	–	21.6	–	22.2	–	23.4	–	**24.0**	–	21.1	–	21.4	–
Su et al.	M	–	49.9	–	–	–	**54.9**	–	–	–	46.7	–	–	–	42.6	–	–	–	41.3
Taiwan (1994) [29]	F	–	27.7	–	–	–	27.2	–	–	–	**30.4**	–	–	–	24.9	–	–	–	22.7
Yu et al.	M	–	35.6	–	–	–	**35.4**	–	–	–	34.3	–	–	–	33.3	–	–	–	29.5
H.K. (2017) [31]	F	–	**22.0**	–	–	–	21.8	–	–	–	21.8	–	–	–	19.9	–	–	–	18.6
Han et al.	M	–	46.4	–	–	–	**49.5 **	–	–	–	46.2	–	–	–	41.3	–	–	–	35.7
S. Korean (2009) [35]	F	–	27.4	–	–	–	**28.8**	–	–	–	28.3	–	–	–	27.7	–	–	–	25.0
Kim et al.	M	40.0	–	42.1	–	44.4	–	**44.7**	–	44.1	–	42.4	–	41.1	–	39.1	–	38.2	–
S. Korean (2018) [39]	F	24.2	–	24.3	–	25.8	–	**26.5**	–	25.8	–	25.7	–	25.5	–	24.2	–	23.5	–
Mathiowetz et al.	M	54.9	–	54.8	–	**55.2**	–	54.3	–	53.0	–	49.8	–	51.5	–	45.9	–	40.7	–
USA (1985) [40]	F	31.9	–	33.8	–	**35.7**	–	33.6	–	31.9	–	28.2	–	29.8	–	26.0	–	25.0	–
Hanten et al.	M	54.9	–	53.6	–	52.2	–	53.1	–	52.7	–	**54.5**	–	53.6	–	43.1	–	42.2	–
USA (1999 [36]	F	31.3	–	33.1	–	33.1	–	**33.6**	–	33.1	–	33.1	–	31.8	–	29.5	–	25.4	–
Bohannon et al.	M	53.3	–	53.9	–	52.8	–	53.3	–	**54.1**	–	50.4	–	50.6	–	44.1	–	41.7	–
USA (2006) [33]	F	30.6	–	33.8	–	33.8	–	33.2	–	32.8	–	**33.9**	–	30.9	–	29.9	–	25.9	–
Wang et al.	M	–	–	**49.7**	–	46.5	–	47.1	–	46.7	–	42.8	–	44.0	–	40.7	–	38.4	–
USA (2018) [45]	F	–	–	29.6	–	28.9	–	29.1	–	**29.9**	–	28.8	–	28.2	–	25.1	–	23.6	–
Nilsen et al.	M	–	49.6	–	–	–	**52.8**	–	–	–	50.7	–	–	–	42.6	-	–	–	36.9
Norway (2012) [43]	F	–	29.1	–	–	–	**29.8**	–	–	–	27.8	–	–	–	26.5	-	–	–	19.8
Harkonen et al.	M	–	51.2	–	–	–	54.0	–	–	–	**55.2**	–	–	–	–	–	–	–	–
Finland (1993) [37]	F	–	31.1	–	–	–	**32.5**	–	–	–	31.6	–	–	–	–	–	–	–	–
Harth et al	M	–	56.3	–	–	–	**57.6**	–	–	–	51.6	–	–	–	49.7	–	–	–	50.6
German (1994) [38]	F	–	32.3	–	–	–	**34.0**	–	–	–	32.9	–	–	–	29.0	–	–	–	28.4
Gunther et al.	M	–	53	–	–	–	**54**	–	–	–	**54**	–	–	–	51	–	–	–	45
German (2008) [34]	F	–	32	–	–	–	**33**	–	–	–	32	–	–	–	28	–	–	–	26
Steiber	M	50.7	–	52.4	–	53.6	–	53.4	–	**53.8**	–	52.9	–	50.4	–	49.1	–	46.3	–
German (2016) [44]	F	32.5	–	33.6	–	33.3	–	34.2	–	**34.5**	–	33.4	–	32.2	–	30.0	–	29.0	–
Werle et al.	M	53.9	–	53.0	–	55.0	–	**55.9**	–	54.2	–	51.8	–	50.8	–	53.6	–	47.9	–
Swiss (2009) [46]	F	33.4	–	34.3	–	33.8	–	**35.8**	–	34.0	–	34.1	–	33.7	–	31.9	–	28.7	–
Adedoyin et al.	M	–	**36.3**	–	–	–	35.0	–	–	–	33.6	–	–	–	27.6	–	–	–	22.8
Nigeria (2009) [32]	F	–	25.1	–	–	–	24.5	–	–	–	22.4	–	–	–	24.8	–	–	–	**26.2**

Note: ^a^: M: Male; F: Female; ^b^: Value with bold and underline represents the peak value at that age strata.

**Table 8:** Comparison of lateral pinch strength among countries (unit: kgw).

Studies	Sex ^a^	Age Strata ^b^																	
		20–24	(20–29)	25–29	(25–34)	30–34	(30–39)	35–39	(35–44)	40–44	(40–49)	45–49	(45–54)	50–54	(50–59)	55–59	(55–64)	60–64	(60–69)
This study	M	7.7	–	–	**9.3**	–	–	–	8.9	–	–	–	8.9	–	–	–	8.3	–	–
Taiwan	F	5.1	–	–	5.6	–	–	–	5.3	–	–	–	**5.7**	–	–	–	5.4	–	–
Wu et al.	M	4.9	–	5.6	–	**6.3**	–	4.4	–	4.9	–	4.9	–	4.1	–	5.2	–	4.3	–
Taiwan (2009) [30]	F	3.0	–	3.4	–	3.7	–	2.8	–	3.3	–	3.4	–	3.9	–	3.6	–	**4.2**	–
Han et al.	M	–	10.5	–	–	–	**11.1**	–	–	–	10.5	–	–	–	10.5	–	–	–	8.7
S. Korean (2009) [35]	F	–	6.5	–	–	–	**7.4**	–	–	–	7.1	–	–	–	7.1	–	–	–	7.1
Mathiowetz et al.	M	11.8	–	**12.1**	–	12.0	–	11.8	–	11.6	–	11.7	–	12.1	–	11.0	–	10.5	–
USA (1985) [40]	F	8.0	–	8.0	–	**8.5**	–	7.5	–	7.6	–	8.0	–	8.0	–	7.1	–	7.0	–
Harth et al	M	–	11.7	–	–	–	11.8	–	–	–	11.9	–	–	–	11.1	–	–	–	**13.1**
German (1994) [38]	F	–	8.0	–	–	–	**8.3**	–	–	–	8.0	–	–	–	7.3	–	–	–	7.6
Werle et al.	M	9.8	–	10.1	–	9.9	–	**10.4**	–	10.3	–	9.8	–	9.7	–	10.3	–	9.8	–
Swiss (2009) [46]	F	6.5	–	6.8	–	6.9	–	7.1	–	**7.2**	–	7.1	–	6.9	–	6.8	–	6.7	–

Note: ^a^: M: Male; F: Female; ^b^: Value with bold and underline represents the peak value at that age strata.

**Table 9:** Comparison of palmar pinch strength among countries (unit: kgw).

Studies	Sex ^a^	Age Strata ^b^																	
		20–24	(20–29)	25–29	(25–34)	30–34	(30–39)	35–39	(35–44)	40–44	(40–49)	45–49	(45–54)	50–54	(50–59)	55–59	(55–64)	60–64	(60–69)
This study	M	7.9	–	–	**8.4**	–	–	–	7.7	–	–	–	**8.4**	–	–	–	7.2	–	–
Taiwan	F	5.2	–	–	**5.6**	–	–	–	5.1	–	–	–	5.3	–	–	–	5.2	–	–
Han et al.	M	–	8.2	–	–	–	**9.9**	–	–	–	9.1	–	–	–	8.6	–	–	–	6.9
S. Korean (2009) [35]	F	–	5.7	–	–	–	**6.7**	–	–	–	6.1	–	–	–	5.9	–	–	–	5.7
Mathiowetz et al.	M	12.1	–	11.8	–	11.2	–	**11.9**	–	11.1	–	10.9	–	10.8	–	10.8	–	9.9	–
USA (1985) [40]	F	7.8	–	8.0	–	**8.8**	–	7.9	–	7.7	–	8.1	–	7.9	–	7.3	–	6.7	–
Harth et al	M	–	10.6	–	–	–	10.9	–	–	–	10.8	–	–	–	10.2	–	–	–	**11.5**
German (1994) [38]	F	–	**7.6**	–	–	–	**7.6**	–	–	–	7.2	–	–	–	7.2	–	–	–	6.7

Note: ^a^: M: Male; F: Female; ^b^: Value with bold and underline represents the peak value at that age strata.

## Abbreviations

WRULDs	Work-related upper limb musculoskeletal disorders
WHO	World Health Organization
EAWS	Ergonomic Assessment Worksheet
KIM-MHO	Key Indicator Method-Manual Handling Operation
OCRA	The Occupational Repetitive Actions
CTS	Carpal tunnel syndromes
ASHT	The American Society of Hand Therapists
R^2^	R-squared
CV	Coefficients of variation
BMI	body mass index
